# Endothelial microvascular networks affect gene-expression profiles and osteogenic potential of tissue-engineered constructs

**DOI:** 10.1186/scrt202

**Published:** 2013-05-17

**Authors:** Torbjorn O Pedersen, Anna L Blois, Zhe Xing, Ying Xue, Yang Sun, Anna Finne-Wistrand, Lars A Akslen, James B Lorens, Knut N Leknes, Inge Fristad, Kamal Mustafa

**Affiliations:** 1 Department of Clinical Dentistry, Center for Clinical Dental Research, University of Bergen, Årstadveien 19, Bergen N-5009, Norway; 2 Department of Biomedicine, University of Bergen, Bergen, Norway; 3 Centre for Cancer Biomarkers, The Gade Institute, University of Bergen, Bergen, Norway; 4 Department of Fibre and Polymer Technology, KTH Royal Institute of Technology, Stockholm, Sweden; 5 Department of Pathology, Haukeland University Hospital, Bergen, Norway

**Keywords:** Tissue engineering, Endothelial cells, Mesenchymal stem cells, Copolymer, Osteogenesis

## Abstract

**Introduction:**

A major determinant of the potential size of cell/scaffold constructs in tissue engineering is vascularization. The aims of this study were twofold: first to determine the *in vitro* angiogenic and osteogenic gene-expression profiles of endothelial cells (ECs) and mesenchymal stem cells (MSCs) cocultured in a dynamic 3D environment; and second, to assess differentiation and the potential for osteogenesis after *in vivo* implantation.

**Methods:**

MSCs and ECs were grown in dynamic culture in poly(L-lactide-co-1,5-dioxepan-2-one) (poly(LLA-co-DXO)) copolymer scaffolds for 1 week, to generate three-dimensional endothelial microvascular networks. The constructs were then implanted *in vivo*, in a murine model for ectopic bone formation. Expression of selected genes for angiogenesis and osteogenesis was studied after a 1-week culture *in vitro*. Human cell proliferation was assessed as expression of ki67, whereas α-smooth muscle actin was used to determine the perivascular differentiation of MSCs. Osteogenesis was evaluated *in vivo* through detection of selected markers, by using real-time RT-PCR, alkaline phosphatase (ALP), Alizarin Red, hematoxylin/eosin (HE), and Masson trichrome staining.

**Results:**

The results show that endothelial microvascular networks could be generated in a poly(LLA-co-DXO) scaffold *in vitro* and sustained after *in vivo* implantation. The addition of ECs to MSCs influenced both angiogenic and osteogenic gene-expression profiles. Furthermore, human ki67 was upregulated before and after implantation. MSCs could support functional blood vessels as perivascular cells independent of implanted ECs. In addition, the expression of ALP was upregulated in the presence of endothelial microvascular networks.

**Conclusions:**

This study demonstrates that copolymer poly(LLA-co-DXO) scaffolds can be prevascularized with ECs and MSCs. Although a local osteoinductive environment is required to achieve ectopic bone formation, seeding of MSCs with or without ECs increases the osteogenic potential of tissue-engineered constructs.

## Introduction

In tissue engineering, the reconstruction of bone defects by using stem cells seeded onto biodegradable carrier materials requires timely formation of functional blood vessels. After *in vivo* implantation, complex tissues are dependent on a functional vasculature, not only for cell survival, but also for tissue organization. In several recent review articles, vascularization was highlighted as a major determinant of the potential size of cell/scaffold constructs [1-3]. The osteogenic potential of bone marrow was demonstrated by Friedenstein *et al.*[4] many years ago. It is suggested that mesenchymal stem cells (MSCs) derived from bone marrow differentiate preferentially along the osteogenic lineage [5]. It is well documented that MSCs can differentiate into skeletal cell types, such as osteoblasts (OBs), chondrocytes, adipocytes, and fibroblasts. Various reports have also found that MSCs possess greater differentiation potential, including differentiation into nonmesodermal cell types. For review, see [6].

The establishment of a biological vasculature within a tissue-engineered construct influences differentiation of cells present and, subsequently, development of the tissue [7]. In the process of bone formation, vascular endothelial cells (ECs) are intimately associated with osteogenic cells, and ECs are regarded as important regulators of osteogenic differentiation [8,9]. The addition of ECs to osteogenic cells has been shown to cause increased bone formation in calvarial defects as well as after subcutaneous implantation [8,10]. Prevascularization, in which three-dimensional EC microvascular networks are developed *in vitro* through coculture of ECs and MSCs before *in vivo* implantation, is therefore an approach whereby the phenotype of MSCs delivered to tissue defects might be altered.

The osteoinductive properties of scaffold materials are also important in optimizing constructs for cell-based tissue regeneration. Several authors have shown that various scaffolding materials, including polymer, can induce ectopic bone formation [8,11,12]. *In vitro* findings have also suggested that poly(LLA-co-DXO) scaffolds enhance differentiation of osteogenic cells [13].

In the present study, ECs and MSCs were cocultured onto three-dimensional copolymer scaffolds. The aim was to investigate how the creation of microvascular networks affect the angiogenic and osteogenic gene-expression profiles. A murine model for ectopic bone formation was used to assess further the cellular differentiation and the potential for osteogenesis after *in vivo* implantation.

## Methods

### Cell culture

Human umbilical vein endothelial cells (ECs) were purchased from Lonza (Clonetics, Walkersville, MD, USA). According to the manufacturer’s instructions, ECs were expanded in Endothelial Cell Growth Medium (EGM) (Lonza) containing 500 ml Endothelial Cell Basal Medium and supplements: FBS, 10 ml; BBE, 2 ml; hEGF, 0.5 ml; hydrocortisone, 0.5 ml; and GA-1000, 0.5 ml. From StemCell Technologies (Vancouver, BC, Canada) primary human bone marrow-derived stem cells (MSCs) were purchased, and expanded in MesenCult complete medium (StemCell Technologies). Flow cytometry was performed to investigate the purity of the cells; it was found that >90% of the cells expressed CD29, CD44, CD105, and CD166, whereas <1% expressed CD14, CD34, and CD45. Cells no older than passage five were used, and all cells were cultured at 37°C and 5% CO_2_. To simplify imaging, retroviral transfection of ECs at an early passage with green fluorescent protein (GFP) was performed [14]. Pulmonary artery smooth muscle cells (SMCs) were purchased from Lonza and expanded in Smooth Muscle Growth Medium-2 (SmGM-2) (Lonza), according to the manufacturer’s instructions. MSCs and SMCs grown *in vitro* were stained with mouse anti-human α-smooth muscle actin (α-SMA) (Santa Cruz Biotechnology, Santa Cruz, CA, USA) incubated in a 1:200 dilution for 4 hours at room temperature with Alexa594-conjugated goat anti-mouse IgG as secondary antibody diluted 1:3,000 for 2 hours.

### Preparation of cell-seeded scaffolds

Fabrication of poly(LLA-co-DXO) scaffolds was described thoroughly in previous publications [15,16], and scaffolds seeded with cells for *in vivo* implantation were prepared in a similar way to the one described by Xing *et al.*[10]. In brief, the scaffolds were prewet with MesenCult complete medium (StemCell Technologies) and incubated overnight at 37°C and in 5% CO_2_. Then, 5 × 10^5^ cells were seeded per scaffold, either MSCs alone or MSCs/ECs in a 5:1 ratio. To facilitate distribution of cells, an orbital shaker (Eppendorf, Germany) was used, and cells were allowed to attach overnight before scaffolds were transferred to separate modified spinner flasks (Wheaton Science, Millville, NJ, USA) for 1 week in a dynamic culture system with 50 rotations per minute. MesenCult complete medium without angiogenic or osteogenic supplements was used for both experimental groups. At 1 week, 6-mm discs were punctured from the scaffolds by using a dermal skin puncher and either processed for *in vitro* analysis or implanted *in vivo*.

### Surgical procedures

All animal experiments were approved by the Norwegian Animal Research Authority and conducted according to the European Convention for the Protection of Vertebrates used for Scientific Purposes, with local approval numbers 3029 and 1572. For subcutaneous implantation, 16 nonobese severe combined immunodeficient (NOD/SCID) mice (Gade Institute/Taconic Farms) were used. Animals were aged 6 to 8 weeks at the time of the operation. An intramuscular injection of 20 μl of Rompun (xylazine) (20 mg/ml) (Bayer Health Care, Leverkusen, Germany) and Narketan (ketamine) (Vétoquinol, Lure, France) in a 1:2 ratio was given to anesthetize the animals. On the backs of the mice, a 2.5-cm incision was made, providing sufficient space for subcutaneous implantation of scaffolds. Wounds were closed with Vetbond Tissue Adhesive (*n*-butyl cyanoacrylate) (3M, St. Paul, MN, USA). Animals were euthanized with deep isoflurane (Schering Plough, Kenilworth, NJ, USA) anesthesia and subsequent cervical dislocation at 1 and 3 weeks. Scaffolds were given careful biopsies and fixated.

As a positive control, 12 3-month-old Lewis rats were operated on in a model described previously [10]. In brief, cell/scaffold constructs were implanted into 6-mm calvarial bone defects, and the animals were euthanized and samples retrieved after 8 weeks.

### Real-time RT-PCR

RNA was extracted by using an E.Z.N.A. Total RNA Kit (Omega Bio-Tek, Norcross, GA, USA). Quantification and determination of RNA purity was performed with a Nanodrop Spectrophotometer (ThermoScientific NanoDrop Technologies, Wilmington, DE, USA). A high-capacity cDNA Archive Kit (Applied Biosystems, Carlsbad, CA, USA) was used for the reverse-transcription reaction. Total RNA (1,000 ng) was mixed with nuclease-free water, reverse transcriptase buffer, random primers, dNTP, and MultiScribe reverse transcriptase. Real-time RT-PCR was performed on a StepOne real-time PCR system (Applied Biosystems). Standard enzyme and cycling conditions were used, with cDNA corresponding to 10 ng mRNA in each reaction, prepared in duplicates for each target gene. Taqman gene-expression assays were human ki67, human and mouse CD31, human and mouse vascular endothelial growth factor (VEGF), human and mouse α-SMA, mouse alkaline phosphatase (ALP), mouse osteopontin (OP), and mouse collagen I (COL I). Data analysis was performed with a comparative Ct method with GAPDH as endogenous control.

For superarray analysis of angiogenesis and osteogenesis, Rt^2^ Profiler PCR Arrays (SuperArray Bioscience, Frederick, MD, USA) were used. Rt^2^ PCR array First Strand Kit (SuperArray Bioscience) was used for cDNA synthesis, and PCR was performed on a StepOne real-time PCR system (Applied Biosystems), with Rt^2^ Real-time SyBR Green/Rox PCR mix (SuperArray Bioscience).

### Histologic evaluation

Samples intended for cryosectioning were immediately frozen in O.C.T. tissue-tech (Sakura Finetek, Tokyo, Japan), by using 2-methylbutan ReagentPlus (Sigma-Aldrich, St. Louis, MO, USA) and liquid nitrogen. Cryosectioning was performed on a Leica CM 3050S (Leica Microsystems, Wetzlar, Germany) at −24°C into 8-μm sections. Samples intended for paraffin sectioning were fixated in 4% PFA before embedding. Sections acquired from the middle parts of the samples were stained with CD31 mouse anti-human primary antibody (BD Biosciences, San Jose, CA, USA) or vimentin mouse anti-human primary antibody (Santa Cruz Biotechnology, Santa Cruz, CA, USA) diluted 1:200 in PBS with 5% goat serum. FITC-conjugated goat anti-mouse (Invitrogen) was used as secondary antibody (1:1,000). DAPI was used for nuclear staining (1:3,000) for 2 minutes at room temperature. Double staining with TRITC-conjugated *Ulex europaeus* Agglutinin (UEA-1) and CD31 was performed to evaluate the interaction between implanted human vessels and the host circulation. All vessels were stained with UEA-1 (Sigma-Aldrich) (diluted 1:500) by incubation for 2 hours at room temperature protected from light. Human vessels were stained as previously described. Scaffolds were autofluorescent with DAPI filter.

Staining for α-SMA was done on 5-μm sections of formalin-fixed and paraffin-embedded scaffolds. The sections were deparaffinized with xylene and rehydrated in decreasing concentrations of ethanol to water. Pretreatment of the sections was done by using antigen retrieval at 57°C overnight in Target Retrieval Solution, pH 6.0 (Dako, Glostrup, Denmark) after 8-minute incubation with Dual Endogenous Enzyme-Blocking Reagent (Dako). Sections were incubated for 30 minutes at room temperature with monoclonal mouse anti-human α-SMA (M0851; Dako) diluted 1:200. Rat-anti-Mouse-IgG2a/AP secondary antibody was used, and α-SMA visualized by incubating with the Ferangi Blue Chromogen Kit (Biocare Medical, Concord, CA, USA) for 20 minutes at room temperature. Antibody specificity was validated by using negative-control sections containing mouse vascular tissue and positive-control sections containing human vascular tissue.

Alkaline phosphatase staining was performed with freshly made substrate solution (Sigma-Aldrich) containing 100 m*M* Tris-maleate buffer, 8 mg/ml Naphthol AS-TR, and 2 mg/ml Diazoniumsalt Fast Red Violet LB. Slides were incubated for 2 hours at room temperature, before washing with distilled water and counterstaining with 0.1% fast green. Alizarin Red staining was performed with 2% Alizarin Red powder (Sigma-Aldrich) dissolved in distilled water, with pH adjusted to 4.2 with 0.5% ammonium hydroxide for 20 minutes at room temperature. A graded alcohol and xylol series was applied before mounting with eukitt (O. Kindler, Freiburg, Germany). Alizarin Red staining was quantified from three sections at 10× magnification for each sample, covering the area of the sections. The total sample size was four mice for each group. A common threshold was applied to all images, identifying high-density areas of red staining by using NIS-Elements BR 3.07 (Nikon, Tokyo, Japan) with a resulting quantified total area fraction of calcified nodules. Masson trichrome and HE staining was performed on *in vivo* samples.

A Nikon 80i microscope (Nikon) was used for light microscopy, and confocal images were acquired on a Zeiss LSM 510 Meta (Carl Zeiss, Oberkocken, Germany). All sections stained with fluorescent secondary antibodies were mounted with Prolong Gold Antifade Reagent (Invitrogen) before imaging.

### Statistical analysis

PCR data presented are from six parallel samples. Groups with monocultured MSCs or MSCs/ECs were compared with the independent samples *t* test, in which *P* < 0.05 was defined as a significant difference. SPSS Statistics 19.0 (IBM, Armonk, NY, USA) was applied for statistical processing and analysis.

## Results

**Figure 1:**
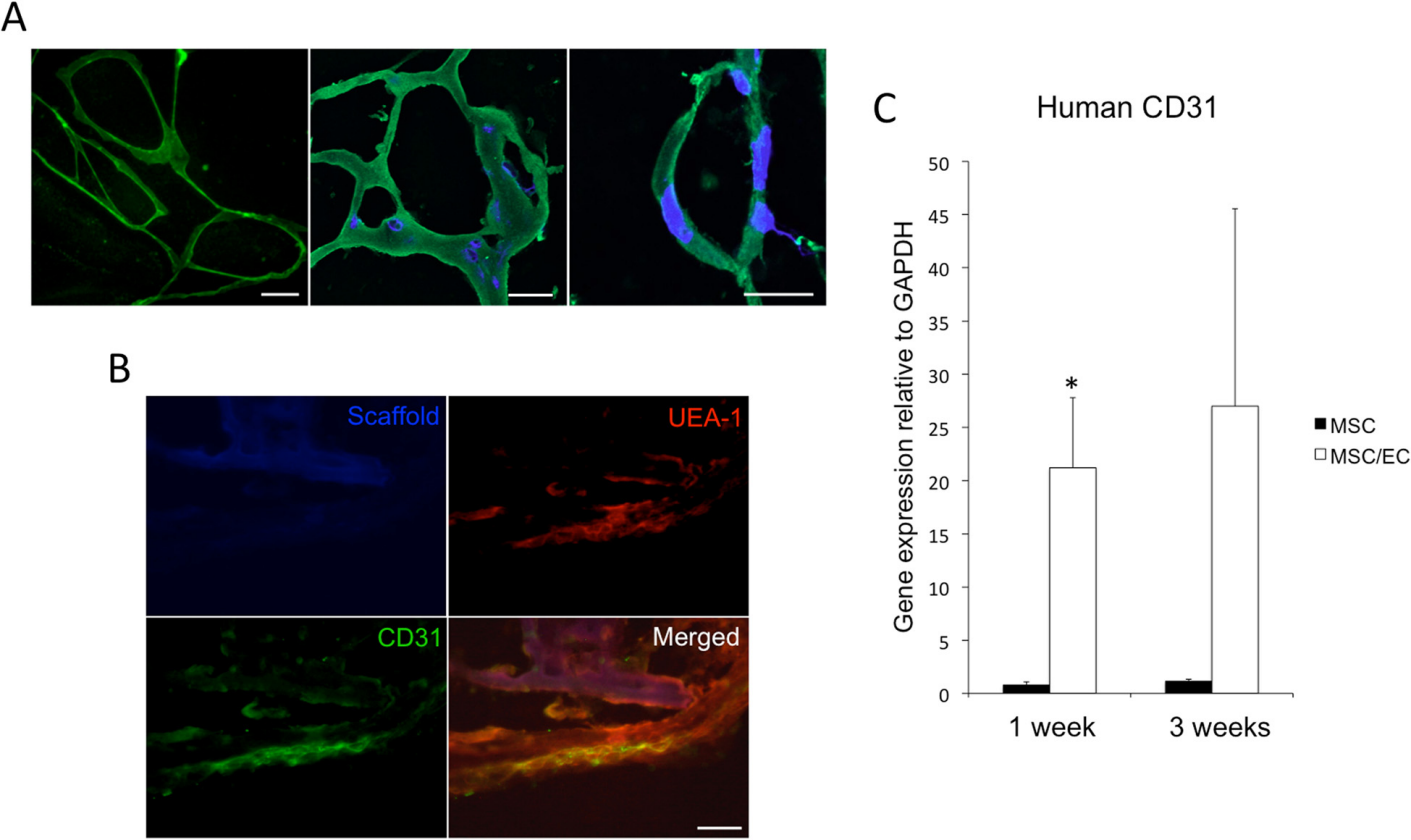
**Development of endothelial cell (EC) microvascular networks in a 3D copolymer scaffold.** (**A**) Left: Light micrograph of GFP-expressing ECs organized in a microvascular network after 1 week of dynamic culture *in vitro* (20×). Scale bar = 50 μm. Middle: Confocal micrograph of ECs stained with human CD31 (green) organized in networks at 1 week *in vivo* (40×). Nuclei were stained with DAPI (blue). Scale bar = 20 μm. Right: Confocal micrograph of tubular CD31^+^ human EC at 1 week *in vivo* (60×). Scale bar = 10 μm. (**B**) Human CD31-positive cells were incorporated with surrounding UEA-1^+^ ECs from the mouse circulation after 1 week of implantation, as demonstrated by double staining (40×). Scale bar = 20 μm. (**C**) The relative expression of human CD31 was higher in MSC/EC constructs after 1 week of implantation, and the expression of human CD31 increased between 1 and 3 weeks. *P < 0.05; *n* = 6.

In EC/MSC constructs, endothelial microvascular networks were observed after 1 week of dynamic culture *in vitro* (Figure 1A, left). Moreover, 1 week after *in vivo* implantation, ECs positive for human CD31 were organized into networks (Figure 1A, middle) and tubular structures (Figure 1A, right). In monocultured MSCs, no human endothelial microvascular structures were detected (not shown). Incorporation of implanted human ECs with the surrounding vascular bed was observed through double staining for human and mouse blood vessels (Figure 1B). As expected, gene-expression levels of human CD31 were higher in cocultured constructs at 1 and 3 weeks of implantation and did not subside during the experimental period (Figure 1C).

**Figure 2:**
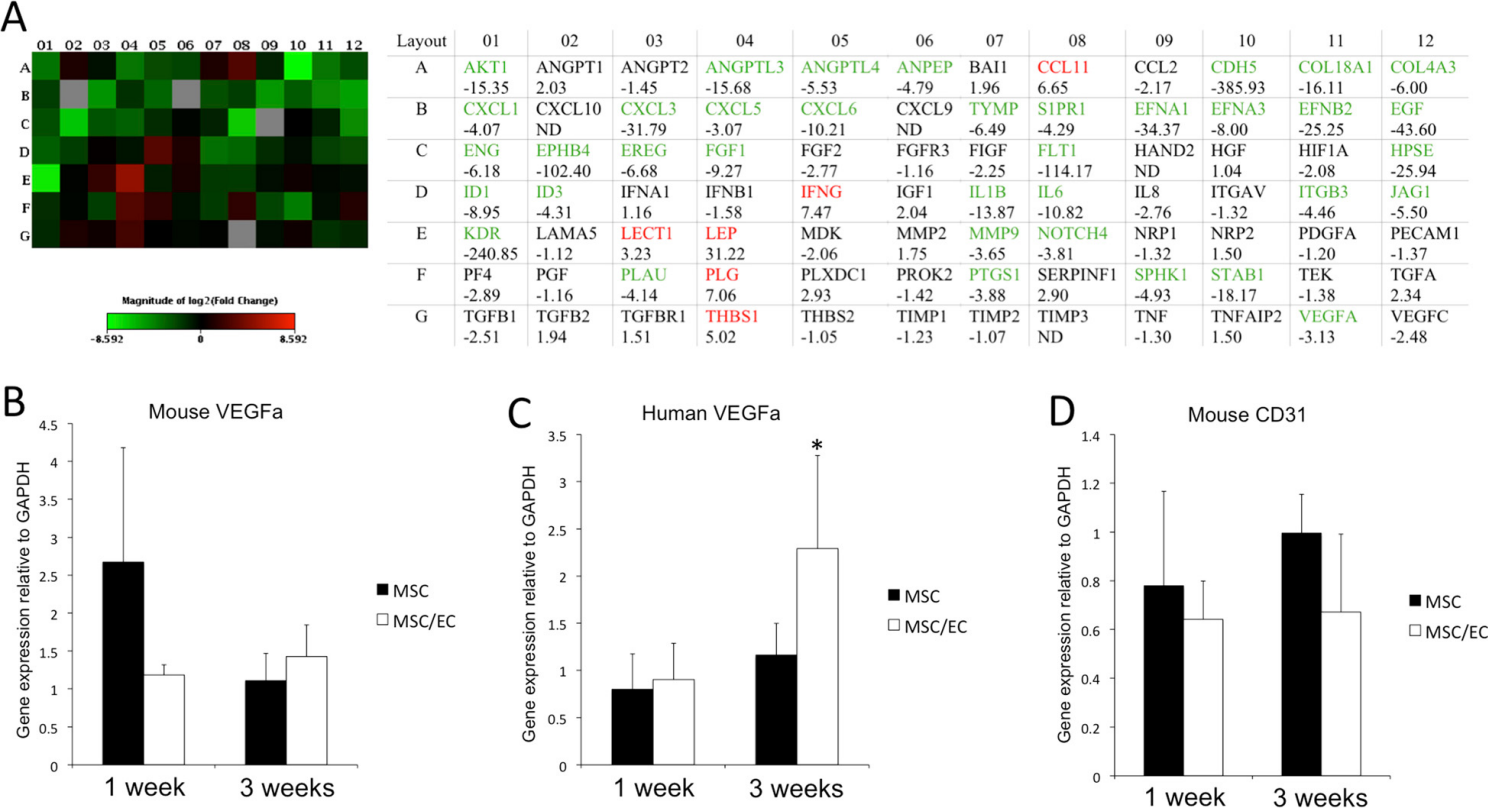
**Angiogenic signaling from mono- and cocultured constructs and the vascular host response.** (**A**) Rt^2^ Profiler Angiogenesis PCR Array after 1 week of dynamic culture *in vitro*. Green squares represent genes expressed higher in monocultured MSCs, and red squares represent genes expressed higher in MSC/EC constructs. Fold changes greater than three are indicated with green or red font, respectively. (**B**) Corresponding VEGF of mouse origin was higher in monocultured constructs at 1 week of implantation. However, the difference was not statistically significant. (**C**) No difference in expression of VEGF from implanted cells was observed at 1 week, whereas an upregulation was found at 3 weeks. *P < 0.05; *n* = 6. (**D**) Expression of mouse CD31 was slightly higher at both time points for monocultured constructs, but the differences were not statistically significant.

Gene-expression profiles were evaluated after 1 week of dynamic culture, before the cell/scaffold constructs were implanted *in vivo*. Evaluation of genes related to angiogenesis showed that in all, 37 genes were downregulated, and six genes were upregulated, with a fold change more than three in the MSC/EC group, compared with monocultured MSCs (Figure 2A). Functional grouping showed that genes related to skeletal development and the extracellular matrix (ECM), were expressed predominantly in monocultured MSCs, whereas higher expression of genes was found related to cell growth and differentiation in cocultures (Table 1). The mRNA expression of VEGF of mouse origin was higher in monocultured constructs at 1 week, although not statistically significantly. At 3 weeks, the expression was similar for both experimental groups (Figure 2B). VEGF from the implanted human cells showed the opposite tendency, with upregulation in cocultured constructs at 3 weeks (Figure 2C). However, when we analyzed the expression of mouse CD31, only minor differences were found during the experimental period, suggesting a similar effect from both constructs on the ingrowth of host ECs (Figure 2D).

**Table 1:** **Functional grouping of osteogenic gene expression after 1 week of dynamic culture *in vitro***

**Gene**	**Full name**	**Fold change**
**Skeletal development**		
*AHSG*	Alpha-2-HS-glycoprotein	−3.7044
*AMBN*	Ameloblastin	−4.9367
*AMELY*	Amelogenin	−4.68
*ENAM*	Enamelin	−2.8164
*STATH*	Statherin	−9.6485
*ALPL*	Alkaline phosphatase	−3.6727
*CALCR*	Calcitonin receptor	−2.9072
*DMP1*	Dentin matrix acidic phosphoprotein 1	−3.9158
*BMP3*	Bone morphogenetic protein 3	−4.5315
*BMP5*	Bone morphogenetic protein 5	−4.9367
*COL2A1*	Collagen type II, alpha 1	−3.373
**Cell growth and differentiation**		
*IGF1R*	Insulin-like growth factor 1 receptor	3.5697
*IGF2*	Insulin-like growth factor 2	4.9403
*TGFB3*	Transforming growth factor beta 3	2.2567
*IGF1*	Insulin-like growth factor 1	2.964
*BMP2*	Bone morphogenetic protein 2	2.476
*GDF10*	Growth differentiation factor 10	2.2778
*TWIST1*	Twist homolog 1	2.1232
*FLT1*	Fms-related tyrosine kinase 1	−8.1751
**Extracellular matrix molecules**		
*MMP10*	Matrix metallopeptidase 10	−5.3112
*CSF2*	Colony-stimulating factor 2	−4.9367
*CSF3*	Colony-stimulating factor 3	−4.9367
*ITGAM*	Integrin alpha M	−3.8558
*COL14A1*	Collagen type XIV, alpha 1	2.7336
*CTSK*	Cathepsin K	2.019

**Figure 3:**
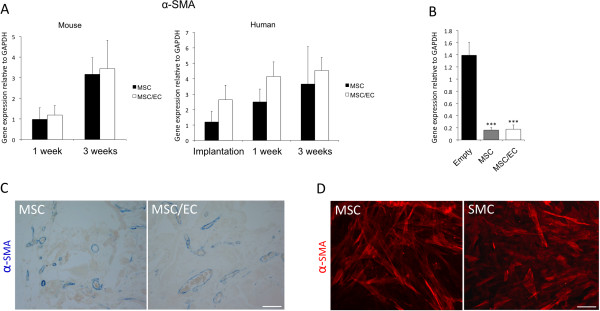
**Expression of perivascular α-smooth muscle actin of mouse and human origin.** (**A**) Left: Expression of mouse α-SMA was upregulated at 3 weeks *in vivo* compared with 1 week. However, intergroup differences were not found. Right: Expression of human α-SMA was upregulated in coculture *in vitro* and at both time points *in vivo*, although the differences were not statistically significant. (**B**) The expression of α-SMA was strongly downregulated for both cell/scaffold constructs at 3 weeks when compared with empty control scaffolds. ***P < 0.001; *n* = 6. (**C**) Positive staining for human α-SMA (20×) surrounding functional blood vessels was observed in both experimental groups at 3 weeks. Scale bar = 100 μm. (**D**) *In vitro* evaluation of MSCs showed positive α-SMA staining comparable to SMC (20×). Scale bar = 100 μm.

Perivascular α-SMA was used as a biomarker to assess maturation of developing mouse vasculature into the scaffolds. An upregulation of mouse α-SMA was observed at 3 weeks compared with 1 week, but no difference was detected between the groups (Figure 3A, left). The expression of human α-SMA was higher in the MSC/EC group at 1 week of culture *in vitro*, and at 1 and 3 weeks *in vivo* (Figure 3A, right). However, the differences were not statistically significant. Interestingly, when compared with control scaffolds in which no cells were implanted, a strong downregulation of mouse α-SMA was observed for both mono- and cocultured constructs (Figure 3B). Positive staining for human α-SMA was found in both groups, surrounding functional blood vessels identified by the presence of red blood cells in the lumen (Figure 3C). When evaluating MSCs *in vitro* for expression of α-SMA, positive staining comparable to that of SMCs was observed for cells grown in standard culture conditions, showing that undifferentiated MSCs express α-SMA (Figure 3D). These findings suggest a perivascular role for MSCs in the development of a functional microvasculature *in vivo*.

**Figure 4:**
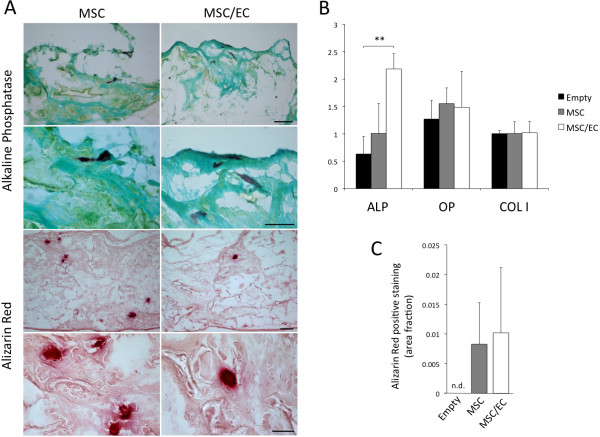
**Osteogenic potential of tissue-engineered constructs after 3 weeks of subcutaneous implantation.** (**A**) Positive staining for alkaline phosphatase (purple) could be observed for both cell/scaffold constructs, as shown at 10× and 60× magnification. Scale bar: upper = 100 μm, lower = 20 μm. Calcified nodules were observed as positive Alizarin Red staining (red) shown at 4× and 40× magnification. Scale bar: upper = 100 μm, lower = 20 μm. (**B**) Gene-expression levels of osteogenic biomarkers alkaline phosphatase (ALP), osteopontin (OP), and collagen I (COL I) were evaluated with real-time RT-PCR, and increased expression of ALP was detected for MSC/EC constructs compared with empty controls. **P < 0.01; *n* = 6. (**C**) Quantification of Alizarin Red staining did not reveal significant differences between MSC and MSC/EC constructs. No positive staining was observed in the control scaffolds without cells; *n* = 4.

The osteogenic potential was evaluated at 3 weeks after implantation, and positive staining for ALP and Alizarin Red was observed for both experimental groups (Figure 4A). Gene expression of ALP was upregulated in cocultured constructs when compared with both empty controls and monocultured constructs. However, only the former comparison was statistically significant. Osteogenic biomarkers OP and COL I were both similarly expressed in the two groups (Figure 4B). Figure 4C shows the total area of calcification as positive Alizarin Red staining, which was higher for both cellular constructs when compared with empty controls, where no positive staining was found. Only minor differences were observed between the two experimental groups.

**Figure 5:**
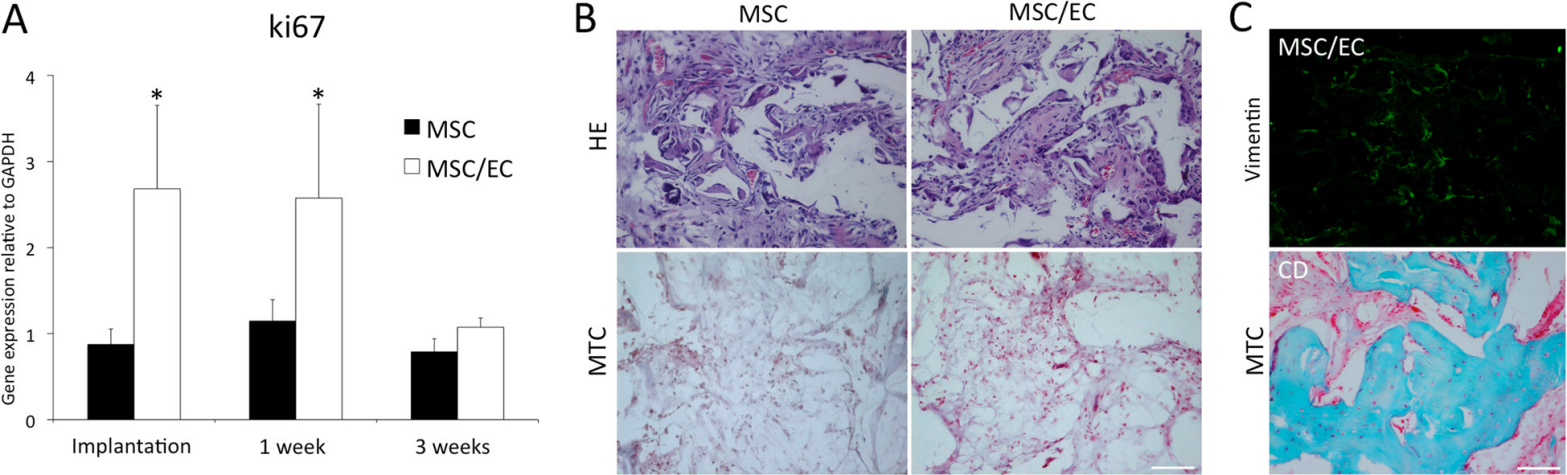
**Survival of implanted cells and the effect of local tissue signaling on osteogenesis.** (**A**) Expression of human ki67 was upregulated at the moment of implantation after 1 week of culture *in vitro*, as well as at 1 week *in vivo*. At 3 weeks *in vivo,* the difference was no longer significant; n = 6. (**B**) Upper: Representative light micrographs (HE staining) of scaffolds penetrated with normal vascular loose connective tissue at 3 weeks subcutaneous implantation (20×). Lower: Masson trichrome staining (20×) could not detect osteoid formation in MSC or MSC/EC constructs at 3 weeks *in vivo*. (**C**) Upper: Representative fluorescent micrograph from the coculture group of cells positive for human vimentin at 3 weeks *in vivo* (20×). Lower: Positive control of MSC/EC implanted in a rat calvarial bone defect showing osteoid formation at 8 weeks (20×). Scale bars = 100 μm.

Human ki67 was used as a biomarker for proliferation of human cells, and the relative gene expression was evaluated before *in vivo* implantation and at 1 and 3 weeks *in vivo*. Human ki67 was significantly upregulated (*P* < 0.05) in the MSC/EC constructs before implantation and at 1 week. At 3 weeks, a difference was still observed, but was not statistically significant (Figure 5A).

After 3 weeks of subcutaneous implantation, scaffolds from both groups exhibited penetration with normal vascularized loose connective tissue (Figure 5B, upper). The mesenchymal cell marker vimentin was used to evaluate the presence of human cells *in vivo,* and cells positive for human vimentin were detected in both groups (Figure 5C, upper). Masson trichrome stain was negative for osteoid formation (Figure 5B, lower), which was evident in cell/scaffold constructs implanted into calvarial bone defects (Figure 5C, lower).

## Discussion

This study evaluated the potential for ECs in combination with MSCs to create microvascular networks in three-dimensional bone tissue engineered constructs. To assess how coculture of MSCs with ECs influenced the phenotype of cells delivered for tissue regeneration *in vivo*, we studied angiogenic and osteogenic gene-expression profiles after 1 week of dynamic culture *in vitro*. The results show that formation of an endothelial microvascular network resulted in upregulated expression of human ki67, a well-described biomarker for cellular proliferation [17]. Various genes involved in cell growth and differentiation, were upregulated in MSC/EC constructs, and the influence of ECs on both angiogenic and osteogenic gene-expression profiles was evident. The results of the *in vivo* experiment showed that the response to the angiogenic signal on ingrowth of CD31-positive cells was similar for both experimental groups, but that implanted human MSCs could support functional blood vessels as perivascular cells. Histologic evaluation showed that generation of ectopic bone in poly(LLA-co-DXO) scaffolds required a local osteoinductive environment. However, increased osteogenic potential was found for both cellular constructs compared with empty controls, and expression of ALP was significantly upregulated in the presence of endothelial microvascular networks.

The potential use of biodegradable polymers as scaffolds for cell-based tissue regeneration was reviewed by Gunatillake and Adhikari [18]. An important attribute of these materials is their chemical versatility, allowing mechanical properties and material degradation to be tailored to specific clinical conditions. Hence these materials are potentially applicable to regeneration of various types of tissue. The response of both OBs and MSCs to the scaffold used in this report has been investigated in several *in vitro* studies [13,15,19]. The results from these studies suggest that the material enhances osteogenic differentiation of both cell types. Our results show that the osteogenic stimulatory effects of the scaffold material and the cellular interactions are not sufficient to induce ectopic bone formation in NOD/SCID-mice. However, several other authors have used polymer scaffolds to deliver osteogenic growth factors and then induce ectopic bone formation [20,21]. MSC/polymer constructs, cultured with or without osteogenic stimulatory conditions before implantation, have also been investigated for their osteogenic potential, with successful generation of ectopic bone [8,22].

Complex tissues depend on functional blood vessels for cell survival as well as for tissue organization after *in vivo* implantation. Prevascularization, in which endothelial cell microvascular networks are developed *in vitro*, have been attempted with different cells and materials. Microvascular networks were created by using MSC/EC coculture spheroids, in which limited functionality was found *in vivo*[23]. Asakawa *et al.*[24] were able to generate a three-dimensional tissue by using dermal fibroblasts as supporting cells for developing endothelial microvascular networks, whereas Yu *et al.*[25] seeded ECs with OBs in poly-ϵ-caprolactone and hydroxyapatite scaffolds, and subsequently demonstrated enhanced osteogenesis in rat long-bone defects. Thus, for development of a *de novo* microvasculature, ECs depend on supporting cells, a role that can be undertaken by several cell types [26,27]. Our results show that a poly(LLA-co-DXO) scaffold can support the formation of EC/MSC microvascular networks in three dimensions, suggesting that prevascularized tissue regeneration with this material is feasible.

In the present study, we cocultured cells for 1 week before *in vivo* implantation and found altered gene-expression profiles with reference to both angiogenesis and osteogenesis. Genes related to skeletal development and the ECM were highly expressed in monocultured MSCs. These results are in agreement with an earlier study by Fuchs *et al.*[28], showing that the expression of osteogenic markers from OBs was downregulated in the presence of ECs in 4-week cultures. Long-term incubation *in vitro* is beneficial when studying the mineralization process, but might be considered less practical for clinical applications and have less potential for regenerating bone [29]. Although an overview of osteogenic and angiogenic gene-expression profiles were generated in the present work, functional evaluations of candidate targets might have been of interest to assess further the effect from regulation of individual genes. Evaluations on tissue development were made on the gene, protein, and morphologic level, to determine the effect of the paracrine signal from all biologic factors delivered by the tissue-engineered constructs.

Murine models of ectopic bone formation are widely used to evaluate osteogenic potential in bone-tissue engineering. By using a subcutaneous mouse model to compare implantation of MSCs and OBs, Tortellini *et al.*[30] reported that MSCs enhanced vascularization through increased recruitment of host ECs. MSCs are known to secrete multiple paracrine factors stimulating EC migration and wound healing [31], and this might be downregulated when ECs are already present in the construct. In the present study, angiogenic gene expression was higher in monocultured MSCs at the moment of implantation, suggesting that increased angiogenic stimulation could be delivered to the host bed from MSCs alone. With ECs already present, paracrine signals to attract and activate ECs might be less relevant in the coculture system.

An initial response from the host circulation was also found as higher expression of mouse VEGF at 1 week, when compared with cocultured constructs. The opposite regulation of human VEGF was observed, with upregulation in the coculture group at the end of the experimental period. These findings might be interpreted as cell/scaffold constructs containing ECs having a stronger ability to maintain a proangiogenic signal after implantation. However, the expression of mouse CD31 was not notably different for the two experimental groups, suggesting a similar vascular host response with regard to the presence of ECs. The total number of blood vessels was not quantified, but no obvious difference could be observed.

Recruitment of perivascular cells with subsequent production of basement membrane proteins are key events in the maturation of developing vasculature [32]. The α-SMA originated from mouse cells was similarly expressed on the mRNA level in MSC and MSC/EC constructs, with an expected increase for both groups between 1 and 3 weeks, as scaffolds were increasingly penetrated with tissue. MSCs in cell/scaffold constructs responded to the presence of ECs by upregulating the expression of α-SMA at all time points, although the differences were not statistically significant. *In vitro* evaluation showed that α-SMA was expressed also in undifferentiated MSCs, whereas the results from the *in vivo* experiment showed that adding ECs resulted in further upregulation of α-SMA. However, the ability of MSCs to function as perivascular cells for developing vessels did not depend on the coimplantation of ECs. Gene expression of α-SMA was strongly downregulated for both constructs when compared with empty controls, suggesting less demand for transcription of α-SMA after implantation of MSCs. Furthermore, α-SMA-positive cells surrounding functional blood vessels were found well distributed in the connective tissue for both experimental groups. In addition to α-SMA, positive staining of human vimentin was demonstrated, showing that implanted MSCs were a viable part of the connective tissue.

Kaigler *et al.*[8] studied dermal microvascular endothelial cells and MSCs seeded on poly(lactic co-glycolic acid) scaffolds implanted subcutaneously in immunodeficient mice. No significant difference was observed in the number of total blood vessels at 2 and 4 weeks, but in line with our findings, a higher percentage of human-derived vessels were found in MSC/EC constructs. At 4 weeks, the area of bone as a percentage of the total tissue area was as high as 35% in the MSC/EC group. In the present study, we cultured cells for 1 week before implantation *in vivo* for 3 weeks, but no osteoid formation was detectable within the constructs. We used MSCs at a lower passage, and this might have influenced the differentiation stage of cells at implantation and, subsequently, the osteogenic potential. In addition, the difference in density of cells with regenerative potential should be considered in the interpretation of the results. When evaluating the expression of osteogenic biomarkers, we found an upregulation of ALP for MSC/EC-constructs compared with both monocultured MSCs and empty control scaffolds. These results are in accordance with those of Xue *et al.*[9] with a two-dimensional coculture MSC/EC model. Compared with monocultured MSCs, at 5 days, multiple osteogenic genes from the cocultured MSC/EC exhibited downregulation, with the exception of ALP [9]. The proposed mechanism for endothelial influence on osteogenic differentiation was therefore that ECs promoted maintenance of MSCs at a proliferative stage rather than inducing terminal differentiation. Although full penetration of tissue within scaffolds was confirmed at the end of the experiment in the present work, longer observation periods might have been of interest to follow further the remodeling and maturation of the tissue.

Positive Alizarin Red staining was found in both cell/scaffold constructs, but not in scaffolds implanted without cells. The majority of the area within the material was filled with connective tissue, with interspersed calcified nodules. The total area of positive staining was not significantly different for the two experimental groups. Both MSCs and MSC/EC thus enhanced the osteogenic potential of poly(LLA-co-DXO) scaffolds after ectopic implantation.

## Conclusions

The results demonstrate that by using a construct comprising MSCs and ECs seeded onto poly(LLA-co-DXO) scaffolds, we can create three-dimensional microvascular networks *in vitro* and sustain them *in vivo*. Furthermore, human MSCs can serve as perivascular cells in the development of functional blood vessels, independent of implanted ECs. The presence of endothelial microvascular networks leads to altered angiogenic and osteogenic gene expression on implantation, and seeding of MSCs with or without ECs increases the osteogenic potential of tissue-engineered constructs.

## Abbreviations

α-SMA: α-smooth muscle actin; ALP: Alkaline phosphatase; COL I: Collagen I; EC: Endothelial cell; ECM: Extracellular matrix; GFP: Green fluorescent protein; MSC: Mesenchymal stem cell; NOD/SCID: Non-obese severe combined immunodeficient mouse; OB: Osteoblast; OP: Osteopontin; poly(LLA-co-DXO): Poly(L-lactide-co-1,5-dioxepan-2-one); SMC: Smooth muscle cell; VEGF: Vascular endothelial growth factor.

## Competing interests

The authors declare that they have no competing interests.

## Authors’ contributions

TOP, ALB, ZX, YX, KNL, IF, and KM conceived and designed experiments. TOP, ALB, ZX, YX and YS performed experiments. TOP, ALB, ZX, and KM analyzed data. TOP, ALB, KNL, IF, and KM wrote the paper. AFW, LAA, JBL and KM contributed reagents, materials and analytical tools. All authors read and approved the final manuscript.
